# Supplementary material for: COZOID: contact zone identifier for visual analysis of protein-protein interactions
Source: BMC Bioinformatics. 2018 Apr 6;19:125. doi: 10.1186/s12859-018-2113-6 (PMC5889581; doi:10.1186/s12859-018-2113-6)
Supplement: Supplementary file 2 — Software build. Executable binary file of the software tool. (ZIP 118,784 kb) [file 12859_2018_2113_MOESM2_ESM.zip › cozoid/platform/docs/testng-6.5.1-javadoc.zip!/org/testng/ITestResult.html]

ITestResult (TestNG)


---


|  |  |  |
| --- | --- | --- |
| **Overview** \| **Package** \| **Class** \| **Tree** \| **Deprecated** \| **Index** \| **Help** | |  |
| **PREV CLASS**   **NEXT CLASS** | **FRAMES**    **NO FRAMES**     **All Classes** |  |
| SUMMARY: NESTED \| FIELD \| CONSTR \| METHOD | DETAIL: FIELD \| CONSTR \| METHOD |  |


---


## org.testng Interface ITestResult

**All Superinterfaces:**: java.lang.Comparable<ITestResult>, IAttributes, java.io.Serializable

---

``` public interface ITestResult extends IAttributes, java.lang.Comparable<ITestResult> ```

This class describes the result of a test.

**Since:**
:   May 2, 2004

**Version:**
:   $Revision: 721 $, $Date: 2009-05-23 09:55:46 -0700 (Sat, 23 May 2009) $

**Author:**
:   Cedric Beust, May 2, 2004

---

| **Field Summary** | |
| --- | --- |
| `static int` | `FAILURE` |
| `static int` | `SKIP` |
| `static int` | `STARTED` |
| `static int` | `SUCCESS` |
| `static int` | `SUCCESS_PERCENTAGE_FAILURE` |


| **Method Summary** | |
| --- | --- |
| `long` | `getEndMillis()` |
| `java.lang.String` | `getHost()` |
| `java.lang.Object` | `getInstance()`             The instance on which this method was run. |
| `java.lang.String` | `getInstanceName()` |
| `ITestNGMethod` | `getMethod()` |
| `java.lang.String` | `getName()` |
| `java.lang.Object[]` | `getParameters()` |
| `long` | `getStartMillis()` |
| `int` | `getStatus()` |
| `IClass` | `getTestClass()` |
| `ITestContext` | `getTestContext()` |
| `java.lang.String` | `getTestName()`             If this result's related instance implements ITest, returns its test name, otherwise returns null. |
| `java.lang.Throwable` | `getThrowable()` |
| `boolean` | `isSuccess()` |
| `void` | `setEndMillis(long millis)` |
| `void` | `setParameters(java.lang.Object[] parameters)` |
| `void` | `setStatus(int status)` |
| `void` | `setThrowable(java.lang.Throwable throwable)` |

**Methods inherited from interface org.testng.IAttributes**

`getAttribute, getAttributeNames, removeAttribute, setAttribute`

**Methods inherited from interface java.lang.Comparable**

`compareTo`

**Field Detail**

### SUCCESS

```
static final int SUCCESS
```

**See Also:**: Constant Field Values

---


### FAILURE

```
static final int FAILURE
```

**See Also:**: Constant Field Values

---


### SKIP

```
static final int SKIP
```

**See Also:**: Constant Field Values

---


### SUCCESS\_PERCENTAGE\_FAILURE

```
static final int SUCCESS_PERCENTAGE_FAILURE
```

**See Also:**: Constant Field Values

---


### STARTED

```
static final int STARTED
```

**See Also:**: Constant Field Values


**Method Detail**

### getStatus

```
int getStatus()
```

:   **Returns:**: The status of this result, using one of the constants above.

---


### setStatus

```
void setStatus(int status)
```

---


### getMethod

```
ITestNGMethod getMethod()
```

:   **Returns:**: The test method this result represents.

---


### getParameters

```
java.lang.Object[] getParameters()
```

:   **Returns:**: The parameters this method was invoked with.

---


### setParameters

```
void setParameters(java.lang.Object[] parameters)
```

---


### getTestClass

```
IClass getTestClass()
```

:   **Returns:**: The test class used this object is a result for.

---


### getThrowable

```
java.lang.Throwable getThrowable()
```

:   **Returns:**: The throwable that was thrown while running the method, or null if no exception was thrown.

---


### setThrowable

```
void setThrowable(java.lang.Throwable throwable)
```

---


### getStartMillis

```
long getStartMillis()
```

:   **Returns:**: the start date for this test, in milliseconds.

---


### getEndMillis

```
long getEndMillis()
```

:   **Returns:**: the end date for this test, in milliseconds.

---


### setEndMillis

```
void setEndMillis(long millis)
```

---


### getName

```
java.lang.String getName()
```

:   **Returns:**: The name of this TestResult, typically identical to the name of the method.

---


### isSuccess

```
boolean isSuccess()
```

:   **Returns:**: true if if this test run is a SUCCESS

---


### getHost

```
java.lang.String getHost()
```

:   **Returns:**: The host where this suite was run, or null if it was run locally. The returned string has the form: host:port

---


### getInstance

```
java.lang.Object getInstance()
```

:   The instance on which this method was run.

---


### getTestName

```
java.lang.String getTestName()
```

:   If this result's related instance implements ITest, returns its test name, otherwise returns null.

---


### getInstanceName

```
java.lang.String getInstanceName()
```

---


### getTestContext

```
ITestContext getTestContext()
```

:   **Returns:**: the `ITestContext` for this test result.


---


|  |  |  |
| --- | --- | --- |
| **Overview** \| **Package** \| **Class** \| **Tree** \| **Deprecated** \| **Index** \| **Help** | |  |
| **PREV CLASS**   **NEXT CLASS** | **FRAMES**    **NO FRAMES**     **All Classes** |  |
| SUMMARY: NESTED \| FIELD \| CONSTR \| METHOD | DETAIL: FIELD \| CONSTR \| METHOD |  |


---
